# Portuguese version of the EUROPEP questionnaire: contributions to the psychometric validation

**DOI:** 10.1590/S1518-8787.2016050006259

**Published:** 2016-09-22

**Authors:** Hugo Roque, Ana Veloso, Pedro L Ferreira

**Affiliations:** I Programa de Pós-Graduação em Psicologia. Escola de Psicologia. Universidade do Minho. Braga, Portugal; II Departamento de Psicologia Aplicada. Escola de Psicologia. Universidade do Minho. Braga, Portugal; III Centro de Estudos e Investigação em Saúde. Faculdade de Economia. Universidade de Coimbra. Coimbra, Portugal

**Keywords:** Surveys and Questionnaires, Translations, Psychometrics, Self Care, Quality of Health Care, Patient Satisfaction, Reproducibility of Results, Validation Studies

## Abstract

**OBJECTIVE:**

To assess the construct validity and reliability of the Portuguese version of the European Task Force on Patient Evaluation of General Practice Care questionnaire.

**METHODS:**

We applied the Portuguese version of the European Task Force on Patient Evaluation of General Practice Care to 392 users of 20 Family Health Units from the North of Portugal. The validity of the construct was evaluated by exploratory factor analysis, with the Principal Axis Factoring method, by orthogonal rotation (varimax procedure), by the Kaiser normalization criteria (eigenvalue ≥ 1). The factorability of the data matrix was verified by the Kaiser-Meyer-Olkin and Bartlett’s sphericity test. We estimated the reliability by the indicator of internal consistency Cronbach’s alpha. To analyze the correlations between satisfaction and loyalty, we used the Pearson correlations. The predictor effect of satisfaction on loyalty was analyzed by simple linear regression.

**RESULTS:**

Satisfaction presented five robust and well individualized dimensions – medical care, nursing care, clinical secretariat services, accessibility, and organization of services – with alpha values between 0.86 and 0.97, good levels of internal consistency. The loyalty showed alpha value of 0.72, considered a reasonable internal consistency. The satisfaction was predictive of loyalty.

**CONCLUSIONS:**

The Portuguese European Task Force on Patient Evaluation of General Practice Care questionnaire is a robust and reliable instrument to measure the satisfaction and loyalty of users of the Family Health Units.

## RESUMO

**OBJETIVO:**

Avaliar a validade de construto e fiabilidade da versão portuguesa do questionário *European Task Force on Patient Evaluation of General Practice Care*.

**MÉTODOS:**

Foi aplicada a versão portuguesa do *European Task Force on Patient Evaluation of General Practice Care* a 392 utentes de 20 Unidades de Saúde Familiar do norte de Portugal. A validade do construto foi avaliada por análise fatorial exploratória, método Factoração de Eixo Principal, por meio da rotação ortogonal (procedimento varimax), pelo critério de normalização de Kaiser (valor próprio ≥ 1). A fatoriabilidade da matriz dos dados foi verificada por meio do *Kaiser-Meyer-Olkin* e do teste de esfericidade de Bartlett. A fiabilidade foi estimada pelo indicador de consistência interna alfa de Cronbach. Para analisar as correlações entre a satisfação e a lealdade, utilizou-se as correlações de Pearson. O efeito preditor da satisfação na lealdade foi analisado por meio de regressão linear simples.

**RESULTADOS:**

A satisfação apresentou cinco dimensões robustas e bem individualizadas – cuidados médicos, cuidados de enfermagem, serviços de secretariado clínico, acessibilidade e organização dos serviços – com valores de alfa entre 0,86 e 0,97, bons níveis de consistência interna. A lealdade apresentou valor de alfa de 0,72, considerada consistência interna razoável. A satisfação foi preditora da lealdade.

**CONCLUSÕES:**

O questionário *European Task Force on Patient Evaluation of General Practice Care* português é um instrumento robusto e fiável para medir a satisfação e lealdade dos utentes das Unidades de Saúde Familiar.

## INTRODUCTION

Quality in health is currently a requirement for all those involved in health care. The quality indicators have been developed in the hospital context and their use quickly spread to primary health care. From an initial focus primarily on scientific and technical aspects, they evolved into the consideration of relational and satisfaction aspects of users^20^. Their usefulness can extend to the own goals of health services. In fact, the received feedback allows to introduce actions of correction and improvement to increase the levels of satisfaction of users^13^ ^,^ ^22^.

Satisfaction is an individual perception linked to the achievement of expectations or satisfaction of a need, by the individual or with intervention of others, whose evaluation is carried out from the person’s perspective. Regarding primary health care, the satisfaction is equivalent to the well-being of users, manifested in their opinion about the quality of the obtained services^24^. It is a dynamic process influenced by individual, psychological, and sociocultural factors, among others. Because it is a multidimensional concept, the satisfaction concerning primary health care can feature an assessment of several aspects, such as accessibility, user-health professional relationship, infrastructure, and health service results^7^ ^,^ ^9^. Thus, to improve the provided services, it is essential to approach the maximum satisfaction of the requirements and needs of users. For this, it is necessary to listen to the views of users on how the services contributed to improve or solve their health state^22^ ^,^ ^24^. This concept of involvement and active participation of users in the provision of health care came from the USA, being later adopted in Europe. Health professionals and users perceive differently the results of the services provided in health. For this reason, the contribution of the user on the quality of services provided is essential. As active agents, the users share their negative and positive experiences and their needs and expectations, contributing to a response system that controls, identifies, and corrects the health system deficits^4^ ^,^ ^27^. Additionally, users’ satisfaction increasingly occupies an important place in assessing the quality, aware of their valorization as active agents and consumers, because it was found that their satisfaction is related to therapeutic adherence and with results achieved and effectiveness of interventions, contributing to change health behaviors^1^ ^,^ ^3^ ^,^ ^19^ ^,^ ^23^.

Thus, it is necessary to engage the population in the construction of instruments for measuring satisfaction, which will allow understanding the expectations of users. The evaluation of services must be multidimensional, considering specific aspects that influence the overall satisfaction. It is necessary to appeal to strict qualitative studies and methods that allow the inclusion of the users’ perspective in the construction processes and their results (questionnaires, tests, interview guides etc.). And the possible negative consequences must also be contemplated, as negative rating or expression of negative expectations, i.e., the respondents must have the opportunity and own space to express their ideas freely^18^ ^,^ ^21^ ^,^ ^24^ ^,^ ^27^.

The European Task Force on Patient Evaluation of General Practice Care (EUROPEP) questionnaire is widely used to evaluate the satisfaction of users with the primary health care, focusing on their perspective of final consumers and active agents in the therapeutic process. This instrument has been prepared using a comprehensive approach, which includes analyses of other investigations and theoretical concepts (literature analysis), interviews with users, and validation studies. The questionnaire aims to evaluate five dimensions: (1) doctor-patient relationship (e.g.: ease with which patients feel comfortable to tell their problems); (2) medical care (e.g.: clinical examination done by the doctor); (3) information and support (e.g.: explanation of the medicines, treatments, and examinations prescribed); (4) organization of services (e.g.: preparation on what to expect of hospital care, other experts, or other care workers); (5) accessibility (e.g.: ease to make an appointment in the family health units (FHU))^2^ ^,^ ^8^ ^,^ ^10^ ^,^ ^11^ ^,^ ^14^ ^-^ ^16^ ^,^ ^26^.

In this context, the aim of this study was to assess the construct validity and reliability of the Portuguese EUROPEP questionnaire.

## METHODS

The study was conducted at the FHU from the districts of Braga, Porto, and Viana do Castelo, which have 172 of the 353 FHU operating in Portugal^25^. From the invited FHU, 20 agreed to participate in this study.

Participants filled out the questionnaires in person in the waiting rooms of the FHU, while waiting for the appointment or after they have been met. The filling occurred in the self and hetero-report format, the latter only on users with low level of education or no education. The collection took place between October 2013 and January 2014. 432 questionnaires were collected, from which 40 were eliminated because they had blank answers or maximum quotation on all items, totaling 392 valid questionnaires.

The 392 users evaluated were aged between 14 and 84 years old, with average of 41.7 years (SD = 15.9). The majority was female (77.3%), married (57.4%), and has already used the FHU services more than once (98.2%). Last year, the participants used the FHU services, on average, 4.7 times (SD = 5.0). The evaluated users were enrolled at the FHU between zero and seven years, with average of 3.9 years (SD = 1.6). Only 13.0% of participants responded in the hetero-report format.

We used the Portuguese version of the EUROPEP, adapted by Ferreira et al.^11^ This instrument features 37 items that assess the level of satisfaction from the perspective of users. The items are rated on *likert* type scale of five points (0 = bad; 4 = excellent). The EUROPEP questionnaire contains, still, three items related to the loyalty of the user toward the FHU^5^ ^,^ ^17^, rated from “0 = disagree a lot” to “4 = agree a lot”.

The construct validity was evaluated by exploratory factor analysis, with the Principal Axis Factoring method, by orthogonal rotation (varimax procedure), by the Kaiser normalization criteria (eigenvalue ≥ 1). The factorability of the data matrix was verified by the Kaiser-Meyer-Olkin and Bartlett’s sphericity test. We estimated the reliability by the indicator of internal consistency Cronbach’s alpha. We used the Pearson correlations to analyze the correlations between satisfaction and loyalty, and simple linear regression to verify the predictive effect of satisfaction on loyalty.

This project was approved by the Ethics Committee of the Regional Administration of North Health, Portugal. All participants signed the informed consent form in accordance with the Helsinki Declaration and the Convention of Oviedo.

## RESULTS

### Satisfaction

The correlation matrix analysis showed significant correlations (p < 0.001) with values between r = 0.24 and r = 0.78. There were no items excessively or perfectly correlated, and it may be affirmed that there are no problems of multicollinearity and singularity, respectively^12^. The analysis of commonalities showed values between 0.46 and 0.87, with average of 0.65, reflecting that 65.0% of the variance associated to items is common or shared. We verified factorability in the data matrix, because the value of Kaiser-Meyer-Olkin (KMO) was greater than 0.60 (KMO = 0.96) and the Bartlett’s sphericity test was significant (p < 0.001).

The internal consistency, estimated by the Cronbach’ alpha, presented high value (0.97) to the full scale, and no items changed, significantly, this value.

The exploratory factor analysis extracted five factors that explain 65.4% of the variance: medical care (31.4%; α = 0.97), organization of services (10.6%; α = 0.89), accessibility (9.9%; α = 0.86), nursing care (7.1%; α = 0.87), and clinical secretariat services (6.4%; α = 0.92).

In Table 1, we present the values of the factor saturation of each item, percentage of total variance, internal consistency of each factor, and the commonalities (h^2^) regarding the satisfaction of users.

**Table 1:** Factor structure and internal consistency of satisfaction.

Satisfaction items	Factor					h^2^

	1	2	3	4	5	
1. Make you feel you have time during the appointment.	0.772					0.671
2. Interest shown by your personal situation.	0.815					0.751
3. Ease with which you felt comfortable to tell your problems.	0.723					0.603
4. How you were involved in the decisions about the care that the doctor provided you.	0.802					0.698
5. How the doctor listened to you.	0.792					0.681
6. Confidentiality of information about your process.	0.589					0.506
7. How the quick relief of your symptoms was provided to you.	0.748					0.669
8. Help you received to make you feel well enough to perform your daily tasks.	0.766					0.694
9. Attention given to your problems.	0.793					0.707
10. Clinical examination done by the doctor.	0.751					0.667
11. Offering of disease prevention services.	0.649					0.466
12. Explanation about medication, treatments, and tests prescribed.	0.768					0.701
13. The way how you were informed about your symptoms and illness.	0.775					0.699
14. Help received to face emotional problems related to your health condition.	0.788					0.683
15. Support received to understand why it is important to follow the advice of your doctor.	0.744					0.686
16. Knowledge about what was said and done in previous contacts on this family health unit.	0.666					0.619
17. Preparation on what to expect of hospital care, other experts, or other health care providers.	0.658					0.599
18. Time devoted to you by the nursing staff.				0.771		0.741
19. Explanations given by nurses on procedures and care performed.				0.844		0.865
20. Time devoted to you in the administrative service.					0.733	0.850
21. The way how you were informed when you had requested information to the clinical secretariat.					0.684	0.804
22. Competence, courtesy, and kindness of the medical staff.	0.586					0.623
23. Competence, courtesy, and kindness of the nursing staff.				0.542		0.594
24. Competence, courtesy, and kindness of the clinical secretariat staff.					0.618	0.770
25. Support, in general, received from the staff at this FHU, besides the doctors.		0.480				0.687
26. Ease in making an appointment that suits you in this FHU.			0.580			0.578
27. Possibility to talk on the telephone for this FHU.			0.763			0.670
28. Possibility to talk on the telephone with the family doctor.			0.683			0.606
29. The opening hours of this FHU.			0.482			0.567
30. Waiting time in the waiting room.			0.492			0.463
31. Freedom of choice of health professional and the possibility of second opinion.	0.460					0.685
32. Overall comfort of this FHU.		0.718				0.651
33. The cleaning of the facilities of this FHU.		0.746				0.482
34. Home services provided by this FHU.		0.539				0.639
35. Overall organization of the services offered by this FHU.		0.580				0.514

Percentage of variance	31.40	10.58	9.92	7.12	6.39	
α	0.97	0.89	0.86	0.87	0.92	

FHU: Family Health Unit

For the retention of items on each scale, the following criteria have been fixed: (1) saturation ≥ 0.40 of each item in the hypothetical factor and only in one single factor; (2) the final factor solution explaining at least 50.0% of the total variance; (3) consistency between the factor solution and items that constitute each factor; and (4) each factor being represented by at least three items^6^ ^,^ ^12^. We removed two items in the sequence of the retention criteria: “The respect with which you were treated and how your privacy was maintained” and “The quickness with which the urgent health problems have been resolved”, because they had a factor loading < 0.40 and similar factor loadings in more than one factor.

In addition to the above procedure, we also performed the exploratory factor analysis by principal component method and maximum likelihood with oblique rotation. These showed similar results, with differences only in the order of the factors.

### Loyalty

The correlation matrix analysis showed significant correlations (p < 0.001), with values between r = 0.35 and r = 0.62. There were no items excessively or perfectly correlated, and it may be affirmed that there are no problems of multicollinearity and singularity, respectively^12^. The analysis of commonalities showed values between 0.25 and 0.76, with average of 0.50, reflecting that 50.0% of the variance associated to items is common or shared. We verified factorability in the data matrix, because the value of KMO was greater than 0.60 (KMO = 0.63) and the Bartlett’s sphericity test was significant (p < 0.001).

The exploratory factor analysis extracted one factor – loyalty – that explains 50.2% of the variance with α = 0.72.

In Table 2, we present the values of the factor saturation of each item, percentage of total variance, internal consistency of the factor, and the commonalities (h^2^) regarding the loyalty. For the retention of the items, we fixed the same criteria used in satisfaction. All items fulfilled the criteria.

**Table 2:** Factor structure and internal consistency of loyalty.

Items of loyalty	Factor	h^2^
1. This FHU meets the needs of users (e.g., of children, older adults, and people with disabilities).	0.497	0.247
2. Strongly recommend this FHU to my friends.	0.869	0.755
3. I do not see any reason to go to another FHU.	0.709	0.503

Percentage of variance	50.15	
α	0.72	

FHU: Family Health Unit

### Relation Satisfaction/Loyalty

The Pearson correlations show significant positive associations (p < 0.01) between satisfaction and its dimensions and loyalty. The highest value was in overall satisfaction, showing that high values of satisfaction are associated with high values of loyalty (r = 0.40; p < 0.01).

The simple linear regression showed that the overall satisfaction of users explains 16.0% of the variance of loyalty (R^2^aj = 0.16; p < 0.001) (F_1;390_ = 74.07; p < 0.001), and, the higher the levels of overall satisfaction, the higher the levels of loyalty with the FHU.

## DISCUSSION

The results show that the items are good indicators of the constructs intended to measure – satisfaction and loyalty – and that the factors are properly individualized. Regarding satisfaction, the results show five robust dimensions, which allow a fairly broad understanding of the users’ satisfaction. Some of these dimensions – medical care, accessibility, and organization of services – find parallels in other validations of the EUROPEP questionnaire^2^ ^,^ ^8^. However, the remaining dimensions are not found in other studies, which can be attributed to the fact that the Portuguese version include more items than the original one. This increase of items meets the reality of the Portuguese primary health care. As for reliability, this instrument features good internal consistency. And, still, there were no problems about the singularity and multicollinearity.

Regarding loyalty, this dimension presented acceptable internal consistency and the items that constituted it compose a robust measure of the construct. Loyalty was positively associated with overall satisfaction and all its dimensions. And, as verified in other studies^5^ ^,^ ^17^, satisfaction is a predictor of loyalty and explains 16.0% of its variance.

In short, the Portuguese EUROPEP questionnaire is a robust and reliable instrument to measure the satisfaction of users by their own perspective.

This study had as its main limitation the apparent sample homogeneity – all FHU belong to the North of the Country – and future studies could appeal to a more heterogeneous sample, involving FHU from the North to the South of the Country. The results show that this is a robust instrument; but it contains many items, being a long questionnaire to apply. A shortened version would be more practical and economical to use in the FHU.
